# A Deep-Learning Approach to ECG Classification Based on Adversarial Domain Adaptation

**DOI:** 10.3390/healthcare8040437

**Published:** 2020-10-27

**Authors:** Lisha Niu, Chao Chen, Hui Liu, Shuwang Zhou, Minglei Shu

**Affiliations:** 1 Shandong Artificial Intelligence Institute, Qilu University of Technology (Shandong Academy of Sciences), Jinan 250353, China; niulisha0717@gmail.com (L.N.); chench@sdas.org (C.C.); liuhui@sdas.org (H.L.); zhoushw@sdas.org (S.Z.); 2 College of Computer Science and Engineering, Shandong University of Science and Technology, Qingdao 266590, China

**Keywords:** ECG classification, deep learning, multi-scale, time features, adversarial domain adaptation

## Abstract

Cardiovascular disease has become one of the main diseases threatening human life and health. This disease is very common and troublesome, and the existing medical resources are scarce, so it is necessary to use a computer-aided automatic diagnosis to overcome these limitations. A computer-aided diagnostic system can automatically diagnose through an electrocardiogram (ECG) signal. This paper proposes a novel deep-learning method for ECG classification based on adversarial domain adaptation, which solves the problem of insufficient-labeled training samples, improves the phenomenon of different data distribution caused by individual differences, and enhances the classification accuracy of cross-domain ECG signals with different data distributions. The proposed method includes three modules: multi-scale feature extraction F, domain discrimination D, and classification C. The module F, constitutive of three different parallel convolution blocks, is constructed to increase the breadth of features extracted from this module. The module D is composed of three convolutional blocks and a fully connected layer, which is to solve the problem of low model layers and low-feature abstraction. In the module C, the time features and the deep-learning extraction features are concatenated on the fully connected layer to enhance feature diversity. The effectiveness of the proposed method is verified by experiments, and the classification accuracy of the experimental electrical signals reaches 92.3%.

## 1. Introduction

Cardiovascular disease has become one of the main diseases threatening human life and health [1]. Clinically, cardiovascular disease is often accompanied by arrhythmia. Serious arrhythmia can lead to sudden death or heart failure [2]. Therefore, timely and accurate detection of arrhythmia is urgent and necessary. Electrocardiogram (ECG), as a physiological signal that characterizes the condition of the heart, is of great significance for the detection and diagnosis of arrhythmia. Moreover, high-precision automatic diagnosis is crucial to the prevention and auxiliary diagnosis and treatment of cardiovascular diseases. The clinical significance of heartbeat classification is that, when a complete ECG signal of a patient is not available and there are only multiple single heartbeats or a small part of the ECG signals, the results of the heartbeat classification will be used to judge the current patient’s heart condition. When an abnormal heartbeat occurs, it is selected as an important heartbeat signal to provide a diagnostic basis for the doctor.

Currently, automatic classification technology mainly includes two categories, namely methods based on computer signal processing technology and methods based on deep learning. The first type of automatic ECG analysis method consists of frequency analysis, decision trees, k-nearest neighbors, support vector machines, artificial neural networks (ANNs), etc. [3,4,5,6,7]. For example, Martis et al. [8] performed an independent component analysis (ICA) on normal, atrial fibrillation, and atrial flutter ECG signals. Compared with the k-nearest neighbor classifier, its classification effect was significantly improved. Acharya et al. [9] proposed a computer-aided diagnosis (CAD) system for the automatic diagnosis of severe arrhythmia, mainly detecting and identifying four kinds of electrocardiogram (normal (N), Atrial Fibrillation(A-Fib), Atrial Flutter(AFL), and ventricular fibrillation(V-Fib)). They extracted entropy features from the ECG signal. After feature selection and extraction, 14 important features were classified through a decision tree. Ye et al. [10] used morphological and dynamic feature methods, which made the detection accuracy reach 86.4%. Shi et al. [11] proposed a hierarchical classification method based on weighted extreme gradient boosting (XGBoost). A large number of features from six categories were extracted from the preprocessed heartbeats. Then, recursive feature elimination was used for selecting features. Afterwards, a hierarchical classifier was constructed in the classification stage. The hierarchical classifier was composed of a threshold and XGBoost classifiers. The final classification accuracy rate reached 92.1%. Although the above traditional classification methods achieved good results, the process of extracting key features from ECG signals and then constructing medical-specific ECG feature vectors is complicated and time-consuming. Secondly, the effect of classification is highly related to the selection of features, which is easily affected by subjective factors, and this method usually shows overfitting [12]. Therefore, they are unreliable in practice, and it is difficult to achieve the expected accuracy and operating efficiency. In contrast, deep-learning methods show more advantages than traditional methods. They can minimize the signal processing and feature extraction process and achieve better classification performance and generalization ability. With the development of deep learning in the computer field, a large number of researchers apply it to the automatic detection and classification of ECG signals. Acharya et al. [12] designed a nine-layer convolutional neural network to realize the automatic diagnosis of arrhythmia and used wavelet transform to reduce the noise. Yildirim et al. [13] used a long short-term memory neural network (LSTM) to identify and classify the ECG. Chu J and Wang Hd et al. [14] first proposed a two-dimensional-convolutional neural network (CNN) for multi-lead ECG signals to extract cross-lead ECG features. The improved multi-lead LSTM network is more convenient than extracting the LSTM features of each lead separately. Features extracted by CNN and LSTM are integrated with traditional features, and binary particle swarm optimization algorithm (BPSO) is used to distinguish and select the features. Finally, a weighted support vector machine is selected as the classifier. Mathews et al. [15] used restricted Boltzmann machines (RBM) and deep belief networks (DBN) to detect supraventricular (S) and ventricular (V) abnormal heartbeats. The sensitivity of the S category was as high as 88.39%. Sellami et al. [16] adopted Resnet to automatically classify ECG signals and proposed a batch-weighted loss to deal with the problem of data imbalance, thus eliminating the complex process of data augmentation. Each input data contained two beats for the model to learn the relevant features better. The overall classification accuracy reached 89%. Wang et al. [17] presented an improved convolutional neural network (CNN) model for accurate classification. The experiment demonstrated that this proposed method had high performance for arrhythmia detection; the accuracy was 99.06%. Golrizkhatami et al. [18] proposed a novel system that exploited multi-stage features from a trained convolutional neural network (CNN) and precisely combined these features with a selection of handcrafted features.

With the development of wearable technology, the acquisition of ECG data is more convenient and efficient. However, due to the high cost of data labeling, many labels are incomplete, and sufficient training data cannot be obtained. There also exist differences in ECG data distribution for individual differences and different data collection sources and methods. The domain adaptive method in transfer learning has advantages in dealing with less labeled data and differences in data distribution. Therefore, the idea of transfer learning is introduced into the classification of ECG signals. With a focus on automatic classification technology based on transfer learning, this paper uses a large number of labeled samples as the source domain data and a small number of unlabeled samples as the target domain data, which improves the interdomain transfer through domain adaptation. Multi-scale feature extractors are helpful to better learn the complex features of signals and improve the model classification performance.

The three main contributions of this paper are listed as follows:

(1) A new ECG signal classification method based on adversarial domain adaptive learning is proposed to solve the problem of insufficient labeled training samples and improve the phenomenon of different data distribution caused by individual differences.
(2) Each component of this method, the F, D, and C modules, is optimized, respectively, to improve the feature diversity and feature abstraction.
(3) Six crucial time features are proposed and concatenated with deep-learning features to increase the richness of the features.

The present paper is organized as follows: Section 2 outlines the ECG dataset and two types of division schemes. Then, input data preprocessing is introduced. The proposed deep-learning structure and training process is described in Section 3. The experiment, evaluation method, and results are presented in Section 4. Finally, Section 5 concludes this paper.

## 2. Materials and Data Preprocessing

**Figure 1:**
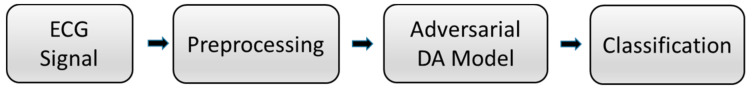
Flowchart of the proposed system. ECG: electrocardiogram. DA: domain adaptation

Figure 1 shows the flowchart of the ECG signal classification task. First, the signal in the ECG record is preprocessed to generate input data, and then, it is passed into the adversarial domain adaptive model for training. The automatically extracted features of the model and the manually extracted time features are fused and input into the classifier to obtain the final classification result. 

### 2.1. ECG Dataset

**Figure 2:**
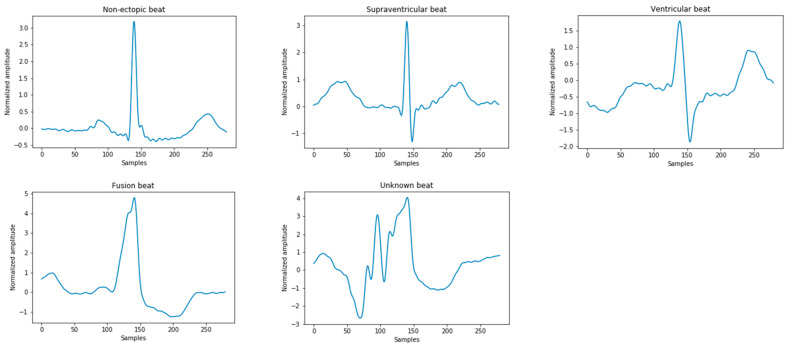
Five types of heartbeat instance diagrams that meet the Association for the Advancement of Medical Instrumentation (AAMI) standards.

The ECG signals used in this study were derived from the Massachusetts Institute of Technology-Beth Israel Hospital (MIT-BIH) arrhythmia database. All records contain the original ECG signals, which are marked by two or more cardiologists [2]. These heartbeats are divided into 15 different types, as shown in Table 1. According to the American National Standards Institute/Association for the Advancement of Medical Instrumentation (ANSI/AAMI EC57:2012) [19], the above 15 types of arrhythmia can be divided into five categories in Figure 2, namely: normal heartbeat (N), supraventricular ectopic heartbeat (S), ventricular ectopic heartbeat (V), fusion heartbeat (F), and unknown heartbeat (Q). In the MIT-BIH database, since the normal QRS complex of modified limb lead II (ML II) is usually prominent, this experiment only uses 44 recorded MLII leads to classify the ECG.

The current mainstream dataset division schemes are mainly divided into two types, namely the intrapatient paradigm [20,21,22] and interpatient paradigm [23,24,25]. In order to make the experimental results more authentic and persuasive, the dataset used in this experiment was divided into an interpatient paradigm. The records 102, 104, 107, and 217 were removed [26,27], since they did not contain stimulated beats from the sinus node of the heart. These 44 records were divided into two groups: DS1 and DS2. The ECG record numbers contained in DS1 were 101, 106, 108, 109, 112, 114, 115, 116, 118, 119, 122, 124,201, 203, 205, 207, 108, 109, 215, 220, 223, and 230; the ECG record numbers contained in DS2 were 100, 103, 105, 111, 113, 117, 121, 123, 200, 202, 210, 212,213, 214, 219, 221, 222, 228, 231, 232, 233, and 234. Table 2 summarizes the dataset evaluation paradigm in this experiment. Since the Q-type data is very small and cannot be used as a basis for judging the classification results, it was removed, and only four types of data: N, S, V, and F were used.

### 2.2. Data Preprocessing

**Figure 3:**
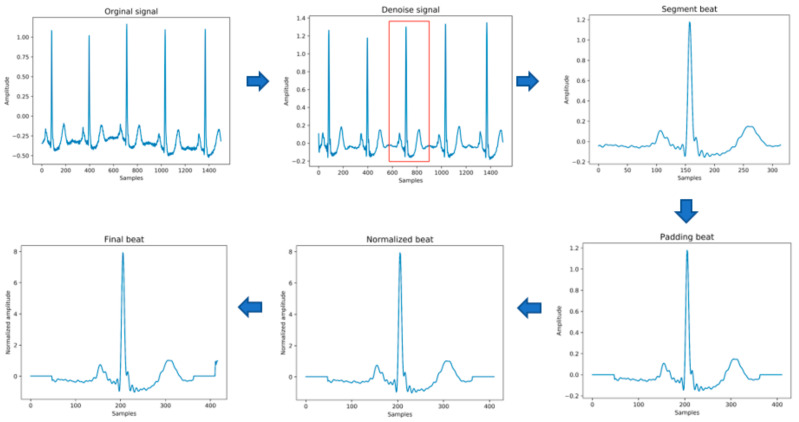
The process of input data generation.

The original data needs to be preprocessed before being passed into the model for training. This stage mainly includes the following steps, as shown in Figure 3 below. In the heartbeat segmentation stage, because the average interval among the adjacent R peaks of different individuals is different, the method of using a fixed number of data points to divide the heartbeat will miss some important signal characteristics of the heartbeat. Therefore, the method of heartbeat segmentation and heartbeat unification proposed below is adopted to solve this problem.

(1) Data denoising: Use the band pass filter F_{band} with a cutoff frequency of (0.5,40) and the discrete wavelet transform (DWT) with a basis function of db6 to eliminate the influence of electrode artifact noise (EMG), muscle artifact noise (MA) and baseline wander noise (BW) on the ECG signal.
(2) Heartbeat segmentation: First, read the R peak position provided in the heartbeat label. Suppose $R_{i}$ is the position of the ith heartbeat R peak. The start position of the heartbeat is $\left\lfloor \frac{1}{2}(R_{i-1}+R_{i})\right\rfloor$, and the end position of the heartbeat is $\left\lfloor \frac{1}{2}(R_{i}+R_{i+1})\right\rfloor$, where $\left\lfloor n\right\rfloor$ represents rounding down to n. Then, the current number of heartbeat sampling points is $H_{i}=\left\lfloor \frac{1}{2}(R_{i}+R_{i+1})\right\rfloor -\left\lfloor \frac{1}{2}(R_{i-1}+R_{i})\right\rfloor +1$.
(3) Heartbeat alignment: After the heartbeats are divided, the number of sampling points $H_{i}$ for each heartbeat becomes different. In order to pass into the deep-learning model, the heartbeats must be aligned. Suppose the number of sampling points after unification is D; if $H_{i}$ is less than D, then fill with zero to D, and if $H_{i}$ is greater than D, then crop to D [28], and the heartbeat after final processing is $H_{f}$. In the experiment of this paper, D is 411.
(4) Data standardization: The aligned heartbeats $H_{f}$ are standardized by the formula $H_{f}=\frac{H_{f}-\mu }{\sigma }$ for the Z-score to eliminate offset and amplitude scaling problems in the signal.
(5) Extraction of time features: Manually extract six time features, including the previous RR intervals of the current heartbeats, the post-RR intervals, the local RR intervals lasting 10s, the average RR intervals of the entire record, and using the formula $\frac{P_{RR}-{\bar{P}}_{RR}}{\max(P_{RR})-\min(P_{RR})}$, to generate the normalized previous RR and normalized post-RR after normalization operation.
(6) Data augmentation: In order to overcome the problem of imbalance in the number of samples in the different categories, the SMOTE algorithm is used to generate different categories of data to balance the dataset.

## 3. Methodology

### 3.1. Model Architecture

Suppose the model is suitable for input samples x∈X and some labels y∈Y and further suppose there are two distributions S (x; y) and T (x; y)—namely, the source domain distribution and the target domain distribution. Both distributions are assumed to be complex and unknown and, furthermore, similar but different. Our goal is to predict the label y from the input x of the target distribution. During the training process, we can access a large number of training samples from the source domain distribution and a small number of target domain distributions {$X_{1},X_{2},X_{3},\ldots X_{N}$}. We define $d_{i}$ as the domain label of the ith data sample to indicate whether $X_{i}$ comes from the source domain or the target domain. If it comes from the source domain, $d_{i}$ = 0; if it comes from the target domain, $d_{i}$ = 1. The implementation principle of the proposed adversarial domain adaptive model is as follows.

**Figure 4:**
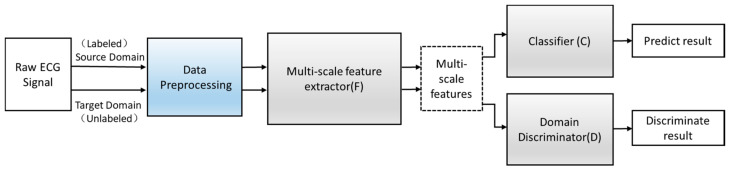
The structure of the adversarial domain adaptation model.

Define an improved adversarial domain adaptive model to predict the corresponding label y∈Y of each input x and their domain label d∈{0,1}. The adversarial domain adaptive model is shown in Figure 4. The constructed adversarial domain adaptive model includes three modules: multi-scale feature extraction F, domain discrimination D, and classification C. 

#### 3.1.1. Multi-Scale Feature Extraction F

**Figure 5:**
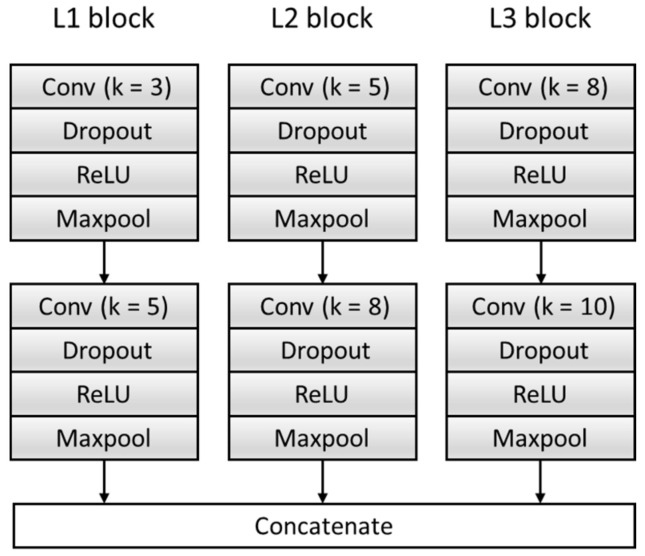
The structure of the feature extractor block. ReLU: Rectified Linear Unit.

To get sufficient features, the F module is improved, and the original single feature extraction structure composed of a single set of convolution blocks is expanded to multiple feature extraction structures composed of three sets of parallel convolution blocks with different convolution kernels. Effectively increase the breadth and richness of the features. Each feature extraction structure consists of two convolutional blocks, and each convolutional block contains a Conv layer, a dropout layer, a Rectified Linear Unit (ReLU) activation function layer, and a Maxpool layer. We transfer the input data $H_{f}$ generated in Section 2.2 into the model to extract three features and then concatenate them as features f—namely, $f=[F_{s1};F_{s2};F_{s3}]$. The specific structure is shown in Figure 5, where k represents the size of the convolution kernel. Table 3 summarizes the architecture of the F Block and output of each layer.

#### 3.1.2. Domain Discrimination D

**Figure 6:**
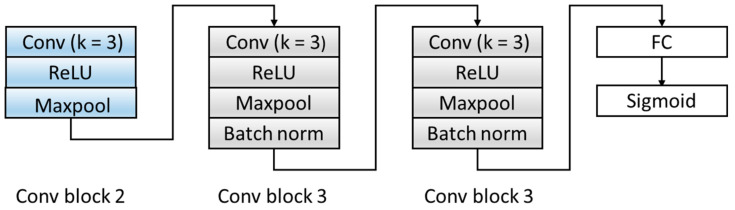
The structure of the domain discriminator block. ReLU: Rectified Linear Unit. FC: fully connect.

Aiming at the problem that the number of original model layers is small and the abstraction of features is low, the D module is optimized, and the original two fully connected layers are expanded into three convolutional blocks and one fully connected layer. The specific structure is shown in Figure 6. The three convolution blocks are as follows: the first convolution block includes: a convolutional layer with a kernel of 3, a ReLU activation layer, and a Maxpool layer. The second and third convolutional blocks have the same structures and are composed of a convolutional layer with a kernel of 3, a ReLU activation layer, a Maxpool layer, and a batch normalization layer. We transferred the features extracted by the F module from the source domain and target domain data to the domain discrimination module D, and we could discriminate the extracted feature source d ∈ {0,1}. Table 4 summarizes the architecture of the D Block and output of each layer.

#### 3.1.3. Classification C

**Figure 7:**
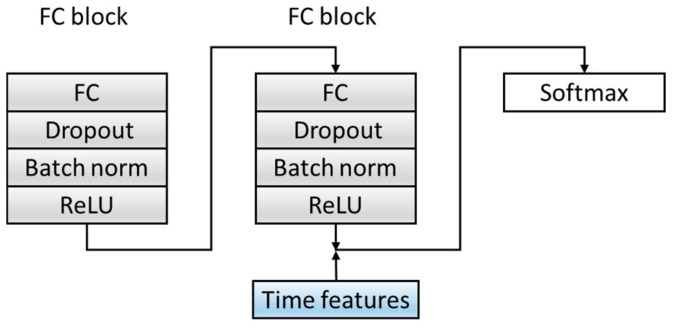
The structure of a classification block. ReLU: Rectified Linear Unit. The convolutional layer and Maxpool layer are represented as Conv_(kernel size)_(kernel number) and Maxpool_(kernel size_(kernel number).

In order to improve the dimensions and richness of the features, the C module is optimized. Before the Softmax layer, the features extracted from the fully connected layer in the source domain data are spliced with the six time features extracted in Section 2.2 as the final feature input to the classifier, which combines time features and deep-learning extraction features, which increases the diversity of the features. The specific structure is shown in Figure 7. Table 5 summarizes the architecture of the C Block and output of each layer.

### 3.2. Training Process

${\left\{(x_{i}^{s},y_{i}^{s})\right\}}_{i=1}^{n_{s}}$ is defined as a labeled instance sample in the source domain Ds, and ${\left\{x_{j}^{t}\right\}}_{j=1}^{n_{t}}$ refers to an unlabeled instance sample in the target domain Dt. F(•) and C(•) are corresponding feature extractors and classifiers. In the learning phase, the goal is to learn a feature extractor F and task classifier C to minimize the expected target loss, align the data distribution of the source domain with the data distribution of the target domain, and reduce the differences among the domains. Among them, the network mappings corresponding to the three modules of F, D, and C are $G_{f}$, $G_{d}$, and $G_{c}$, respectively. The joint loss function $E(\omega_{f},\omega_{c},\omega_{d})$ is introduced, as shown in Equation (1).

(1) $$\begin{array}{ll} E(\omega_{f},\omega_{c},\omega_{d}) & =\sum_{\begin{array}{l} i=1..N\\ d_{i}=0 \end{array}}L_{c}(G_{c}(G_{f}(X_{i};\omega_{f});\omega_{c}),y_{i})\;-\lambda \sum_{i=1..N}L_{d}(G_{d}(G_{f}(X_{i};\omega_{f});\omega_{d}),y_{i})\\ & =\sum_{\begin{array}{l} i=1..N\\ d_{i}=0 \end{array}}L_{c}^{i}(\omega_{f};\omega_{c})-\lambda \sum_{i=1..N}L_{d}^{i}(\omega_{f};\omega_{d}) \end{array}$$

The loss function mainly includes two parts—namely, the classification loss $L_{c}^{}(•,•)$ and the domain discrimination loss $L_{d}^{}(•,•)$. $L_{c}^{i}$ and $L_{d}^{i}$ represent the corresponding loss function calculated in the ith training sample. The $L_{c}^{}$ choose a focal loss instead of using traditional cross-entropy loss. $\omega_{f},\omega_{c},\omega_{d}$ are the parameters of the multi-scale feature extraction module, the parameters of the classification module, and the parameters of the domain discrimination module. λ represents the weight between the two learning objectives. $d_{i}=0$ indicates that the ith sample is a source domain sample. The training process is shown in Formulas (2) and (3).

(2) $$\left({\overset{⌢}{\omega }}_{f},{\overset{⌢}{\omega }}_{c})=\arg\underset{\omega_{f},\omega_{c}}{\min}E(\omega_{f},\omega_{c},{\overset{⌢}{\omega }}_{d}\right)$$

(3) $$\left({\overset{⌢}{\omega }}_{d})=\arg\underset{\omega_{d}}{\max}E({\overset{⌢}{\omega }}_{f},{\overset{⌢}{\omega }}_{c},\omega_{d}\right)$$

**Figure 8:**
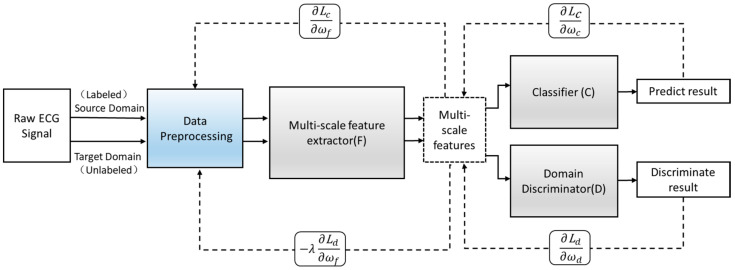
The training process of the adversarial domain adaptation model. $L_{c}$: classifier loss.$\;L_{d}$: discriminator loss. $\frac{\partial L}{\partial \omega }:$ derivative.

The specific training steps are listed as follows:

(1) The first step is to keep the parameters of the domain discrimination module $\omega_{d}$ unchanged and calculated by Formula (2) to maximize the loss of the domain discrimination module to update the parameters of the multi-scale feature extraction module $\omega_{f}$ to obtain the domain-invariant features. In this way, the invariant features can be fully obtained, which can summarize the source domain data and target domain data at the same time. Minimize the loss of the classification module to update the parameters of the classification module $\omega_{c}$ to obtain a classifier that accurately predicts the label. ${\overset{⌢}{\omega }}_{f}$, ${\overset{⌢}{\omega }}_{c}$,and ${\overset{⌢}{\omega }}_{d}$ are the parameter values of the saddle point $\omega_{f}$, $\omega_{c}$, and $\omega_{d}$, respectively.
(2) The second step is to fix the parameters $\omega_{f}$ and $\omega_{c}$, and keep them unchanged. Use Formula (3) to minimize the loss of the domain discrimination module and update the parameters of the domain discrimination module $\omega_{d}$ to obtain a strong discriminator that can distinguish the source of the feature.
(3) The third step is to repeat the operation of the first step, the parameters of the fixed domain discrimination module $\omega_{d}$ are unchanged, and the feature extraction module and the classification module are trained through Equation (2). Use this training process and update the parameters alternately.
(4) In the end, the network maintains a dynamic balance. After reaching the preset number of iterations, the optimal value is obtained, and the optimal model is saved. Input the new heartbeat sample into the saved optimal model to obtain the final classification result. The training process is shown in Figure 8.

## 4. Results and Discussion

### 4.1. Experimental Setting

Based on the MIT-BIH database, experiments were carried out to verify the effectiveness of the proposed method. In terms of data division, all heartbeat samples in DS1 were defined as source domain data, and the heartbeat samples in the first five min of each record in DS2 were regarded as target domain data. The training data included the source domain data and its label and target domain data, and the test data was the entire DS2 data. After training, the optimal model was saved, and all the heartbeat samples of DS2 were transferred to the saved optimal model for testing. In the training process, the grid search method was used to select the optimal parameters from the mass parameters. The entire model was trained using Stochastic Gradient Descent (SGD) optimizer with default parameters and the learning rate of 0.001 at the beginning. Dropout, as a widely used regularization method, was beneficial to the model’s generalizability and was set as 0.2. λ was set to 0.2 to balance the weight of the two classifier parameter updates. The batch size of the training was set to 128 for better updating model parameters.

The experiment was trained on a server equipped with Tesla V100-SXM2 GPU. The dynamic memory of the computer was 32480MiB and the Ubuntu 16.04 operating system. The network was implemented through the Pytorch Advanced Neural Network Application Programming Interface (API).

### 4.2. Performance Metrics

As a multi-class classification problem, it is different from binary classification. For a category, *TP* represents the number of heartbeats that are correctly detected, *TN* represents the number of heartbeats that are not detected correctly, *FP* contains the number of heartbeats of other categories that were classified as this category, and *FN* contains the number of heartbeats of this category that were classified as other categories. Under AAMI’s classification standards, the ratio of Q data is very small (less than 1%). The classification performance of this class has a negligible impact on the overall performance. At the same time, the proportion of S, V, and F classes is much higher (about 10%). These three categories contain most of the arrhythmia. Therefore, we focused on the classification accuracy of the four categories N, S, V, and F.

Following the guidelines provided by the AAMI, three performance indicators were calculated—namely, accuracy (*Acc*), sensitivity (*Sen*), and positive predictive value (*PPV*), as below:

(4) $$Acc=\frac{TP+TN}{TP+TN+FP+FN}$$

(5) $$Sen=\frac{TP}{TP+FN}$$

(6) $$PPV=\frac{TP}{TP+FP}$$

### 4.3. Experiments Results and Discussion

This section is divided into three parts to illustrate the effectiveness and efficiency of the proposed adversarial domain adaptive network. The first part is the algorithm validity experiment. The second part is a comparison of the different models experiment, which verifies the efficiency of the proposed algorithm by changing the feature extraction module and adding time features. The third is a comparison of other paper methods experiment. By comparing with different algorithms, the effectiveness of the proposed algorithm is further validated.

#### 4.3.1. Experiment 1: Algorithm Validity

**Figure 9:**
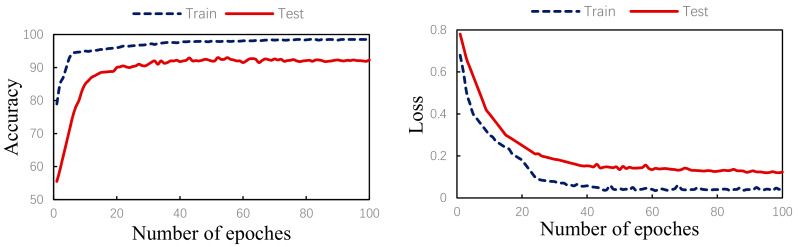
Training/test accuracy and loss curves.

**Figure 10:**
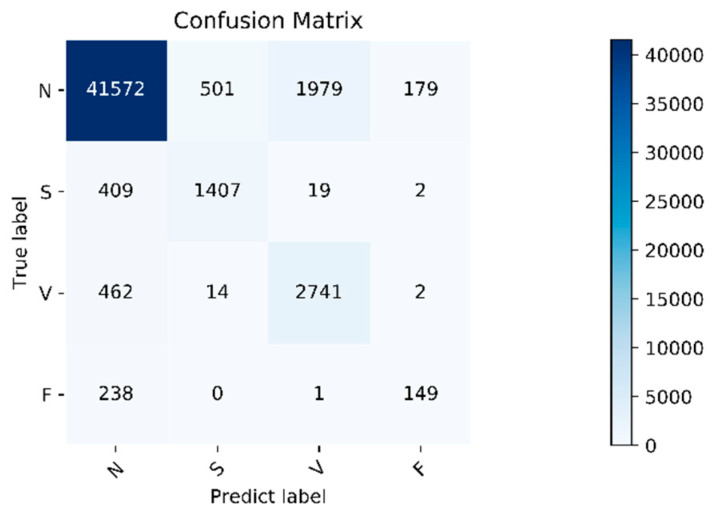
Confusion matrix. Normal heartbeat (N), supraventricular ectopic heartbeat (S), ventricular ectopic heartbeat (V), and fusion heartbeat (F).

The algorithm validity experiment of the paper was proposed. The results of this method were as follows: Figure 9 shows the changes in the loss curve and accuracy curve during the model training and testing process. It can be seen from the figure that the loss of the test set had the same trend as the training set. At the same time, the accuracy of the train set and the test set gradually increased towards the expected direction. There was no sharp decline in the accuracy of the test set, indicating that there was no overfitting. The confusion matrix of the proposed algorithm is shown in Figure 10. It can be seen from the figure that, in order to improve the classification accuracy of the S and V heartbeats, the accuracy of the N heartbeats was sacrificed. Among the N heartbeats, 501 heartbeats were mistakenly classified as S and 1979 heartbeats as Class V. The main reason is the waveform characteristics of some signals.

Class N are similar to those of Class S and V, but the overall, the classification effect is still within an acceptable range. The overall accuracy of the program is 92.3%. For the four categories of N, S, V, and F, the positive predictive value of the system is 97.4%, 73.2%, 57.8%, and 44.9%, respectively. The sensitivities of the four categories are 94.0%, 76.6%, 85.2%, and 38.4%, respectively. Due to the small amount of data in Class F, insufficient training in this category cannot be avoided even using the data augmentation method, so the classification accuracy is slightly lower. Fortunately, the PPV value of class F is relatively high, indicating that not as many other classes are misclassified into Class F. Specific evaluation indicators are shown in Table 6.

#### 4.3.2. Experiment 2: Comparison of Different Models

In order to verify the influence of the multi-scale feature extractor and the addition of time features on the experimental results, the following three models are used for comparison experiments:

(1) Single-scale Model A. Among them, Model A uses three convolutional layers, three pooling layers, and two fully connected layers to build. The kernel size of the convolutional layer is 3.
(2) Model A+RR. The six time features proposed in Section 2.2 are added to the last fully connected layer of Model A, and the stitched overall features are input into the Softmax classifier for classification.
(3) The model proposed in the paper. During the experiment, with the use of the same input data, the comparison of the classification results is shown in Table 7 below.

It can be seen from the table that the first group uses a single-scale Model A compared with the second group with added time features. The second group of models has higher training accuracy. The reason is that the time features are manually obtained based on professional knowledge; the time features are similar to adding expert knowledge to the classifier, so that the accuracy of the classification is effectively improved. Since the waveform characteristics of many signals in class S are similar to those of class N, the biggest difference lies in the disappearance of P waves and the change of RR intervals compared with class N. That is to say, the addition of time features plays a key role in the discrimination of class S heartbeats, and the accuracy of the S class is significantly improved.

Comparing the second group of single-scale models with the third group of multi-scale models, it can be seen that the multi-scale feature extraction module can effectively improve the classification accuracy. This is because the complex network structure can extract the overall characteristics of the signal more comprehensively rather than partial characteristics, and it can also extract more detailed features that are easily overlooked. Table 7 shows that the overall accuracy of the training results is 92.3%, and the positive predictive values of the four types of heartbeats are significantly higher than the second group. Although the recall rates of the S and V categories in the second group of single-scale models are slightly higher, many heartbeats in the N category are classified into the S category, resulting in a significantly lower positive predictive value of the S category, and the overall classification accuracy rate is also declined due to the decreasing accuracy of the N category.

In summary, the classification performance of the multi-scale adversarial domain adaptive model proposed in this paper is better than the other two models.

#### 4.3.3. Experiment 3: Comparison of Other Paper Methods

Table 8 lists the classification results obtained by other papers under the interpatient evaluation scheme. It can be seen that the results obtained by using the proposed model (method) are significantly better than the method proposed by Chazal [23], except for the slightly lower positive predictive values of the N and V types. The method proposed in this paper takes the signal of one lead as the input, and there is no complicated feature extraction process. The difference with that of Chazal is that he uses many domain-specific features extracted from the two-lead ECG signal to construct the classifier. The extracted features are used as the input of the statistical classifier model of each lead signal, and a third classifier is built to integrate the outputs of the first two classifiers. Therefore, their method is computationally expensive and time-consuming; it is not suitable for real-time heartbeat classification on one-lead ECG monitoring equipment.

Compared with Ye et al. [10], the method proposed in this paper does not require a complicated process of manually extracting features, and the overall accuracy and various classification situations are significantly better than Ye’s method.

In Mathews et al. [14], compared with the results of the method proposed in this article, although the sensitivity of the S and F categories are significantly improved, the classification accuracies of the N and V categories are reduced, especially the N category, which only reaches 73.89%. The overall accuracy also reduced to 74.81% due to the decline of the N category. Therefore, the method proposed in this paper showed obvious advantages in the classification accuracy of the N class, V class, and the overall classification accuracy.

In Sellami et al. [16], although the accuracy of Class S, V, and F were higher than that of this paper, the accuracy of class N was significantly lower. In this dataset, N type accounted for 89%, indicating that a large part of the data was misclassified. Compared with the method proposed in paper [16], the classification accuracy of Class N was significantly higher than that of Class A (88.51%), while the classification accuracy of Class S and Class V was slightly lower but also within the acceptable range, and the PPV value of class S and F reached 73.2% and 44.9%, respectively, which were significantly higher than [16] (30.44% and 8.6%).

In Shi [11], compared with the proposed paper, the S, V, and F class classification accuracy was a certain advantage, as opposed to the overall accuracy, and the N class accuracy was slightly less at the same time. Meanwhile, this paper needed to manually extract six categories, 168 signal characteristics, and needed two hierarchical weighted XGBoost classifiers to classify. This undoubtedly increased the complexity and time-consuming characteristics of the method.

## 5. Conclusions

Aiming at the problems of less-labeled training data, different data distributions caused by individual differences and single-feature extraction, a new multi-scale heartbeat classification model based on adversarial domain adaptation was proposed. Each component of this method, the F, D, and C modules, was optimized, respectively. The F module was optimized to extract ECG features of multiple scales from convolution blocks composed of different convolution kernels. The D module was optimized to three convolutional blocks and a fully connected layer, and the C module was enhanced by the time feature and the amplitude feature confusion. The proposed method was effective. The adversarial domain adaptive learning method was used to solve the problem of insufficiently labeled training samples and improve the phenomenon of different data distribution caused by individual differences. The design of the multi-scale feature extractor in module F and the fusion of the time feature and automatic feature in module C can improve the richness and diversity of the extracted data features. Through experiments, the effectiveness of the improved adversarial domain adaptive learning classification model is verified.

Since the classification accuracy of the F class needs to be improved, the primary work in the future is to further optimize the classification effect of the F class. Secondly, we will focus on adaptive learning between different datasets and adaptive learning between different leads.

## Figures and Tables

**Table 1:** A summary table of electrocardiogram (ECG) beats categorized as per the American National Standards Institute/Association for the Advancement of Medical Instrumentation (ANSI/AAMI EC57: 2012) standard. Normal heartbeat (N), supraventricular ectopic heartbeat (S), ventricular ectopic heartbeat (V), fusion heartbeat (F), and unknown heartbeat (Q).

N	S	V	F	Q
Normal	Atrial premature	Premature ventricular contraction	Fusion of ventricular and normal	Paced
Left bundle branch block	Aberrant atrial premature	Ventricular escape		Fusion of paced and normal
Right bundle branch block	Nodal(junctional) premature			Unclassifiable
Atrial escape	Supra-ventricular premature			
Nodal (junctional) escape				

**Table 2:** Interpatient evaluation paradigm of the MIT-BIH arrhythmia database. MIT-BIH: Massachusetts Institute of Technology-Beth Israel Hospital.

Datasets	Number of Heartbeats				Total
	N	S	V	F	
DS1	45824	943	3787	414	50,968
DS2	44213	1836	3219	388	49,656
Total	90,037	2779	7006	802	100,624

**Table 3:** A summary table of the multi-scale feature extractor (F) block. ReLU: Rectified Linear Unit.

Block	Layers	Output Size
	Input (preprocessed ECG signal)	411 × 1
L1 block	Conv_3_16,stride=1Dropout,0.2ReLUMaxpool_2_16,stride=2	204 × 16
L1 block	Conv_5_32,stride=1Dropout,0.2ReLUMaxpool_2_32,stride=2	100 × 32
L2 block	Conv_5_16,stride=1Dropout,0.2ReLUMaxpool_2_16,stride=2	203 × 16
L2 block	Conv_8_32,stride=1Dropout,0.2ReLUMaxpool_2_32,stride=2	98 × 32
L3 block	Conv_8_16,stride=1Dropout,0.2ReLUMaxpool_2_16,stride=2	202 × 16
L3 block	Conv_10_32,stride=1Dropout,0.2ReLUMaxpool_2_32,stride=2	96 × 32
	Concatenate	9408 × 1

The convolutional layer and Maxpool layer are represented as Conv_(kernel size)_(kernel number) and Maxpool_(kernel size)_(kernel number).

**Table 4:** A summary table of the domain discriminator (D) block. FC: fully connect

Block	Layers	Output Size
	Input (the output of the F model)	9408 × 1
Conv block 2	Conv_8_6,stride=1ReLUMaxpool_2_6,stride=2	4691 × 6
Conv block 3	Conv_10_6,stride=1ReLUMaxpool_2_6,stride=2BatchNorm	2336 × 6
Conv Block 3	Conv_10_6,stride=1ReLUMaxpool_2_6,stride=2BatchNorm	1159 × 6
	FC layer (3474,100)	100 × 1
	Sigmoid	2

The convolutional layer and Maxpool layer are represented as Conv_(kernel size)_(kernel number) and Maxpool_(kernel size)_(kernel number).

**Table 5:** A summary table of the C block.

Block	Layers	Output Size
	Input (the output of the F model)	9408 × 1
FC block	FC(9408,100)Dropout,0.2BatchNormReLU	100 × 1
FC block	FC(100,10)Dropout,0.2BatchNormReLU	10 × 1
	Add(10,6)	16 × 1
	Softmax	4

The convolutional layer and Maxpool layer are represented as Conv_(kernel size)_(kernel number) and Maxpool_(kernel size)_(kernel number).

**Table 6:** The results of the proposed method. Accuracy (Acc), sensitivity (Sen), and positive predictive value (PPV).

Type	Sen (%)	PPV (%)	Acc (%)
N	94.0	97.4	91.8
S	76.6	73.2	98.0
V	85.2	57.8	93.5
F	38.4	44.9	99.0
Average	73.6	68.3	95.5

**Table 7:** The results on the proposed method compared with the single-scale method. RR: six time features.

Model	Acc	N (Sen, PPV)	S (Sen, PPV)	V (Sen, PPV)	F (Sen, PPV)
Model A	89.0	90.1, 97.6	63.5, 35.7	90.4, 54.6	20.6, 28.4
Model A+RR	91.3	92.5, 97.8	81.9, 59.1	88.8, 56.1	23.7, 43.8
Proposed	92.3	93.9, 97.4	76.6, 73.2	85.1, 57.8	38.4, 44.9

**Table 8:** Comparison of the results between the proposed method and previous works. Normal heartbeat (N), supraventricular ectopic heartbeat (S), ventricular ectopic heartbeat (V), and fusion heartbeat (F).

Method	Year	Acc (%)	N		S		V		F	
			Sen (%)	PPV (%)	Sen (%)	PPV (%)	Sen (%)	PPV (%)	Sen (%)	PPV (%)
Chazal	2004	85.8	86.8	99.1	75.9	38.5	77.7	81.9	68.2	26.5
Ye	2012	86.4	88.6	97.5	60.8	52.3	81.5	63.1	19.5	2.5
Mathews	2018	74.81	73.89	99.66	88.39	33.63	77.24	69.20	73.7	4.67
Sellami	2019	88.34	88.51	98.80	82.04	30.44	92.05	72.13	89.4	8.6
Shi	2019	92.1	92.1	99.5	91.7	46.2	95.1	88.1	61.6	15.2
Proposed	-	92.3	93.9	97.4	76.6	73.2	85.1	57.8	38.4	44.9
